# A NILM load identification method based on structured V-I mapping

**DOI:** 10.1038/s41598-023-48736-8

**Published:** 2023-12-02

**Authors:** Zehua Du, Bo Yin, Yuanyuan Zhu, Xianqing Huang, Jiali Xu

**Affiliations:** 1 Ocean University of China, Qingdao, 266100 China; 2 Pilot National Laboratory for Marine Science and Technology (Qingdao), Qingdao, 266237 China

**Keywords:** Energy science and technology, Engineering

## Abstract

With the increasing number and types of global power loads and the development and popularization of smart grid technology, a large number of researches on load-level non-intrusive load monitoring technology have emerged. However, the unique power characteristics of the load make NILM face the difficult problem of low robustness of feature extraction and low accuracy of classification and identification in the recognition stage. This paper proposes a structured V-I mapping method to address the inherent limitations of traditional V-I trajectory mapping methods from a new perspective. In addition, for the verification of the V-I trajectory mapping method proposed in this paper, the complexity of load characteristics is comprehensively considered, and a lightweight convolutional neural network is designed based on AlexNet. The experimental results on the NILM dataset show that the proposed method significantly improves recognition accuracy compared to existing VI trajectory mapping methods.

## Introduction

In 2021, the domestic electricity consumption of urban and rural residents in China will account for 14.13% of the electricity consumption of the whole society, a year-on-year increase of 7.3% . At present, the energy of thermal power generation is higher than 68% of the total power generation, and the increasing electricity consumption of urban and rural residents will undoubtedly aggravate the global energy crisis and environmental pollution. At the same time, according to the operation information of a single load, users can understand the energy consumption rules of each device and achieve up to 14% power saving^1^. Therefore, with the help of effective energy management solutions and through fine-grained energy monitoring and analysis, improving energy structure and optimizing electricity consumption habits are effective means to improve energy efficiency. The non-intrusive load monitoring method was proposed by Professor Hart of the Massachusetts Institute of Technology^2^, and has received a lot of attention from researchers^3–8^. The NILM method collects power data by installing an intelligent acquisition module at the household end, and analyzes the collected data with the help of statistical learning methods or artificial intelligence technology, so as to realize real-time monitoring of all load usage conditions in the household electricity environment^9^. By analyzing the energy consumption of different devices and the interaction between the grid end and the user end, the optimization of the energy usage structure can be realized.

Non-intrusive load monitoring technology is relative to intrusive load monitoring technology^2^. Intrusive load monitoring technology needs to install sensors for each device in the building to monitor detailed energy consumption data, however, this method undoubtedly has problems such as high cost and complicated installation. Therefore, non-intrusive load monitoring method came into being, which can be divided into event-based NILM and non-event based NILM according to different technical routes^10^. The non-event based NILM method separates the load-level samples from the aggregated signals for analysis. However, due to the linear correlation between partial load signals and the uncertainty of the separation results, it is difficult to decompose the signals through the traditional Blind source separation technology^11^, which requires more external support or other perspectives for load analysis^12,13^, this makes it difficult to analyze the state and energy consumption of a single load; the event-based NILM method continuously monitors the state of the event to confirm the time of the event, and extracts the changing load data for analysis^14–23^. Although the event-based NILM method needs to combine multiple stages to realize load analysis, and each step is crucial to the accuracy of NILM, this method has been widely studied with strong applicability and generalization. The method proposed in this paper is an event-based non-intrusive load identification method.

Non-intrusive load identification can be divided into mathematical optimization and pattern recognition. In the early stage of the development of NILM technology, there were relatively many studies on load type identification based on mathematical optimization methods^2,14,15,24^. Lin et al.^14^ used heuristic algorithm to solve the load decomposition model based on steady state current to identify specific loads and handle simultaneous events, but its identification accuracy was not high. Bergman et al.^15^ assumed that the active power consumed by each electrical device was different, and then ran the improved knapsack algorithm on each edge time to obtain a good identification effect, but did not involve loads with continuous state changes. In general, the model of the mathematical optimization method is essentially an NP-complete problem, and the solution efficiency is a challenge. Moreover, such methods are difficult to meet the needs of high-frequency signals, a large number of load types and high-precision load analysis. The load identification method based on pattern recognition has received extensive attention in recent years^25–27^, especially with the gradual maturity of CNN-based image processing technology and the emergence of NILM recognition methods based on VI trajectory mapping, a large amount of research has started to apply image classification methods to load recognition problems^16–20,23,28–33^. Iksan et al.^28^ proposed the earlier VI trajectory mapping method, which fully expresses the spatial structural relationship between current and voltage through a pixelated trajectory mapping method. Lam et al.^16^ characterized the signal characteristics of household loads through the voltage-current (V-I) trajectory, and used a hierarchical clustering method to construct a classification model. Hassan et al.^20^ expanded and evaluated the V-I trajectory-based appliance load signature to achieve predictive accuracy and robustness in a classification algorithm used to decompose residential overall energy use and predict constituent appliance profiles. Du et al.^30^ abstracted the similarity of voltage-current (V-I) trajectories between loads and proposed to map the V-I trajectories to a grid of cells with binary values to provide graphic signatures for loads. De Baets et al.^33^ mapped the devices represented by VI trajectories to a newly learned feature space created by a siamese neural network, enabling the samples of the same device to form a tight cluster. Then, DBSCAN performs cluster analysis, allowing the method to assign device samples to clusters or label them as “unidentified”. However, it is foreseeable that the image-based load identification method similar to the V-I trajectory needs to map discrete current points and voltage points into a graph to achieve the continuity of the V-I trajectory, which will bring about efficiency problems in the conversion process. In addition, the V-I trajectory method relies on the normalization of current and voltage values, which will lead to high similarity in the characterization of similar electrical appliances, thus bringing difficulties to the identification.

It can be seen from this that the traditional time-series data expression method has limited features that can be extracted from the data-driven network model, and the image-based and V-I trajectory feature expression methods have problems such as loss of amplitude signals and imperfect spatial structure features, which will lead to increased training costs and low accuracy of load identification. Therefore, how to construct a data sample form with richer feature expression and full coverage of feature information to achieve more effective feature expression samples, combined with optimization model, and finally achieve accurate identification of load identification is the key technology that needs to be solved in this paper.

Therefore, the main contribution of this paper is to propose a structured V-I mapping representation for load identification. This method embeds the traditional V-I relative spatial position expression into the thermal representation of V-I maps and establishes sufficient point-to-point correlations in V-I to improve the richness of neural network feature extraction. The effectiveness and superiority of the proposed method are experimentally validated on various constructed datasets.

## Proposed method

The main task of load identification in NILM is to perform feature extraction and classification identification from decomposed unknown devices signals. This method is usually based on event detection and signal decomposition. This paper assumes that the signal to be analyzed is the load-level signal to be identified obtained through the relatively ideal preceding process.

Define the target device to be analyzed as *i*, $i \in \{ 1,2,...,n\}$, where *i* represents the index of the device type, and *n* represents the number of types of target devices. The current data of appliance *i* is ${S_i} = \{ {s_i}(1),{s_i}(2),...,{s_i}(t)\}$, where ${s_i}(t)$ represents the current value of the *i*-th device at time *t*. Similarly, the voltage data of appliance *i* is ${U_i} = \{ {u_i}(1),{u_i}(2),...,{u_i}(t)\}$.

Because there is a fixed periodic law in the load operation process, the periodic data under the ideal stable state is consistent, and the identification of the load type can be simplified to the identification of the load periodic data. Therefore, it is first necessary to decompose the current and voltage data of electrical appliances in a periodic manner. Since the voltage has stable periodic expression characteristics, we can decompose the periodic data of the voltage and extract the current data corresponding to the index to realize the periodic decomposition of load operation data.

Assume that the starting index of the first period data is $t_1$, satisfying ${u_i}({t_1} - 1)*{u_i}({t_1}) \le 0$, and ${u_i}({t_1}) \ge 0$; assuming that the starting index of the second period data is $t_2$, also satisfying ${u_i}({t_2} - 1)*{u_i}({t_2}) \le 0$, and ${u_i}({t_2}) \ge 0$. Then we define $\left[ {{t_1},{t_2}} \right)$ as a complete power frequency cycle, and its corresponding data $U_i^{{T_j}} = \{ {u_i}({t_j}),{u_i}({t_j} + 1),{u_i}({t_j} + 2),...{u_i}({t_{j + 1}} - 1)\}$ and $S_i^{{T_j}} = \{ {s_i}({t_j}),{s_i}({t_j} + 1),{s_i}({t_j} + 2),...{s_i}({t_{j + 1}} - 1)\}$ are a complete voltage and current cycle respectively, where $U_i^{{T_j}}$ represents the *j*-th cycle data of the *i*-th device, ${u_i}({t_j})$ represents the $t_i$-th data of the *i*-th device, and the description of the current is the same. At the same time, we define the length of a period as *l*. For the current and voltage of the whole dataset level, the distribution of data is constrained by the normalization method shown in Eq.(1).

1 $$\begin{aligned} U = \frac{{U - \bar{U}}}{{\sigma (U)}},I = \frac{{I - \bar{I}}}{{\sigma (I)}} \end{aligned}$$

Among them, $\bar{U}$ and $\bar{I}$ represent the mean value of *U* and *I* respectively, and $\sigma$ represents the standard deviation. Define three-dimensional tensor $Q_i^{{T_j}}(d,x,y) = \left\{ {q_i^{{T_j}}(1,1,1),q_i^{{T_j}}(1,1,2),...,q_i^{{T_j}}(3,l,l)} \right\}$, where $d \in \{ 1,2,3\}$ represents the dimension index of the tensor, x and y represent the numerical index under the specified dimension respectively, and $q_i^{{T_j}}$ represents the data corresponding to the *j*-th cycle tensor of device *i*. Then we agree that the first dimension of the tensor is the multiplicative property mapping, which represents the coupling characteristic expression between current and voltage; the second dimension is the additive property mapping, which represents the isotropic characteristic expression between current and voltage; the third dimension is subtractive property mapping, which represents the anisotropic feature expression between current and voltage.

2 $$\begin{aligned} Q_i^{{T_j}}(d,x,y) = \left\{ {\begin{array}{*{20}{c}} {s_i^{{T_j}}(x)*u_i^{{T_j}}(y),} &{} {d = 1} \\ {s_i^{{T_j}}(x) + u_i^{{T_j}}(y),} &{} {d = 2} \\ {\left| {s_i^{{T_j}}(x) - u_i^{{T_j}}(y)} \right| ,} &{} {d = 3} \\ \end{array}} \right. \end{aligned}$$

**Figure 1:**
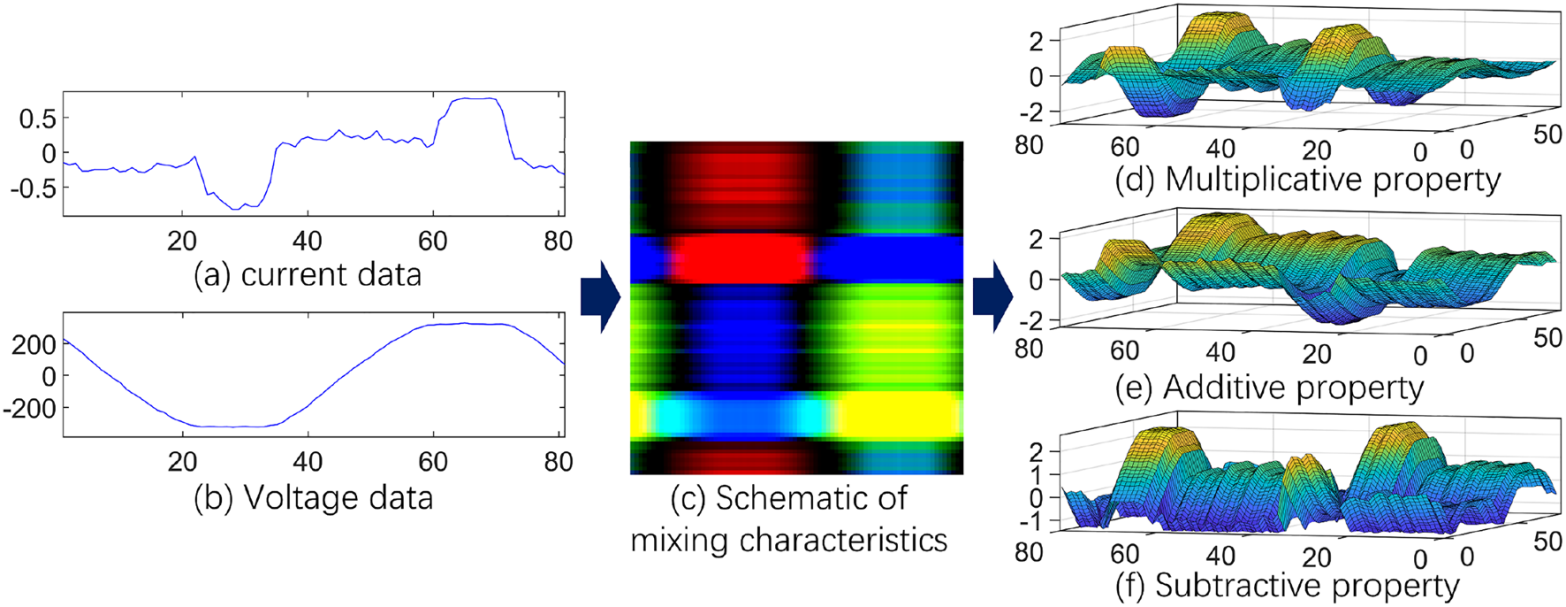
Schematic diagram of structured V-I mapping.

Among them, $x \in \left[ {1,l} \right] ,y \in \left[ {1,l} \right]$. It can be seen from the Eq.(2) that in each dimension of the tensor $Q_i^{{T_j}}$, the current and voltage have established a point-to-point feature expression. Since the samples of the convolutional neural network are input in the form of tensors, this paper combines three different forms of feature expressions into three channels of a tensor as the input of the model for the neural network to extract more comprehensive features from different angles, as shown in Fig. 1.

In Fig. 1, the subgraphs (a) and (b) are the original periodic signals of current and voltage. The correlation mapping between voltage and current is realized through the above method. The implementation of this method does not perform secondary transformation on current and voltage, retains their original information and relative spatial structure relationship, and embedded into the numerical information of tensor elements. In addition, compared with other methods, the implementation process of the method in this paper is simple and convenient for efficient model calculation.

To validate the effectiveness of the proposed structured VI trajectory mapping method, the convolutional neural network constructed in this paper is designed with the AlexNet network^34^ as the prototype structure and combine it with depth-wise separable convolution for lightweight implementation. The AlexNet network structure is simple, so it is more convenient for flexible structure optimization design. And because the network has fewer network layers than other deep networks, so it has more shallow channels, which are suitable for feature extraction of the three independent channels of the structured V-I mapping in this paper.

In addition, in order to achieve targeted feature extraction from three independent channels while reducing the number of parameters and computational costs, this paper introduces a depthwise separable convolution module^35^ to replace the basic convolution modules involved. The basic operation unit of depthwise separable convolution consists of two parts: Depthwise (DW) and Pointwise (PW).

**Figure 2:**
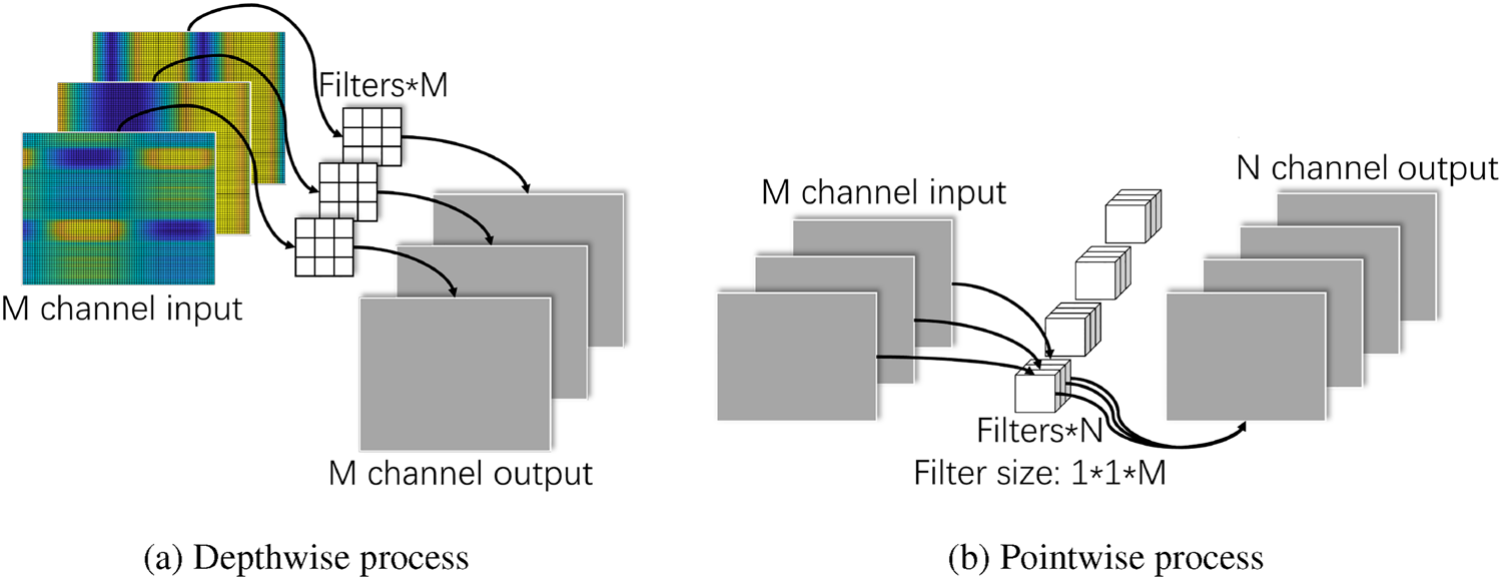
Schematic diagram of process.

Depthwise Convolution designs convolution kernels with the same number of input channels, each convolution kernel is only responsible for one channel, and it extracts the features in the channel and generates the corresponding feature mapping. Therefore, the number of input channels, the number of convolution kernels, and the number of feature map channels generated during the DW process are all M. Through the DW convolution operation process, the feature extraction of the specified channel by the convolution kernel can be realized without being affected by other channel features, as shown in Fig. 2a.

The main purpose of the Pointwise Convolution partial operation is to perform feature weighted combination of the DW partial output. The size of its convolution kernel is 1x1xM, and M is the number of channels in the previous layer. The number of convolution kernels of PW part is consistent with the number of output channels, both of which are N, as shown in Fig. 2b.

Therefore, the AlexNet-DS network structure and parameter settings finally simplified and designed in this paper are shown in Table 1.

**Table 1:** AlexNet-DS network structure.

Layer Index	Structure Setting	Kernel Size	I/O Channel	Stride
1	CBB	3*3	channel-input, 64	2
	MaxPool	2*2	/	2
2	CBB	3*3	64, 192	1
	MaxPool	2*2	/	2
	CBB	3*3	192, 384	1
4	CBB	3*3	384, 256	1
	MaxPool	2*2	/	2
5	Linear	/	6400, 1024	/
	RELU	/	/	/
6	Linear	/	1024, 1024	/
	RELU	/	/	/
	Linear	/	1024, class num	/

## Experiments

### Datasets

In order to construct different power consumption scenarios, better reflect the generalization performance of the proposed method, and be able to define some extreme power consumption environments, this paper collects power consumption datasets and conducts experiments based on the embedded signal acquisition module developed by the laboratory project team. This dataset is adopted at a frequency of 4kHz and contains up to 23 electrical appliance combinations. The sample data and basic operating instructions involved have been uploaded to github(https://github.com/duzehua/DataSet_SEIN_1).

According to the different electrical functions or main electronic components, superclasses and classes are defined. The modeling work in this paper is based on the identification experiments of classes. The main function of superclasses is to control similar electrical appliances in the dataset. Each class contains 5000 cycles of current and voltage data as a training set, and an assigned label. In addition, 500 cycles of samples corresponding to the load are collected as a test set to verify the effectiveness of the method. According to the different components of electrical appliances, the four types of datasets constructed are shown in Table 2.

**Table 2:** Electrical composition and category labels of 4 datasets.

Index	Superclass	Name	Abbreviation	Dataset 1 Label	Dataset 2 Label	Dataset 3 Label	Dataset 4 Label
1	Notebook (NBS)	Notebook1	NB1	0	0	0	N/A
		Notebook2	NB2	1	N/A	1	N/A
2	Refrigerator (RGS)	Refrigerator1	RG1	2	1	2	N/A
		Refrigerator2	RG2	3	N/A	3	N/A
3	Motor (MRS)	ElectricFan1	EF1	4	2	4	N/A
		ElectricFan2	EF2	5	N/A	5	N/A
		Vacuum	VM	6	N/A	6	N/A
		Ventilator	VR	7	N/A	7	N/A
4	Resistiveheater (RHS)	ElectricHeater1	EH1	8	3	8	0
		ElectricHeater2	EH2	9	N/A	9	1
		ElectricOven	EO	10	N/A	10	2
		ElectricKettle1	EK1	11	N/A	11	3
		WaterDispenser	WD	12	N/A	12	4
		ElectricKettle2	EK2	13	N/A	13	5
		ElectricCooker	EC	14	N/A	14	6
5	LCD(MTS)	Monitor	MT	15	4	15	N/A
		Television	TV	16	N/A	16	N/A
6	AirPurifier (APS)	AirPurifier	AP	17	5	N/A	N/A
7	MainFrame (MFS)	MainFrame	MF	18	6	N/A	N/A
8	InductionCooker (ICS)	InductionCooker	IC	19	7	N/A	N/A
9	Oxygenerator (OGS)	Oxygenerator	OG	20	8	N/A	N/A
10	Tablet PC (TBS)	Tablet1	TB1	21	9	17	N/A
		Tablet2	TB2	22	N/A	18	N/A

Note: N/A means that the dataset does not contain the data of this type of electrical appliances.

**Figure 3:**
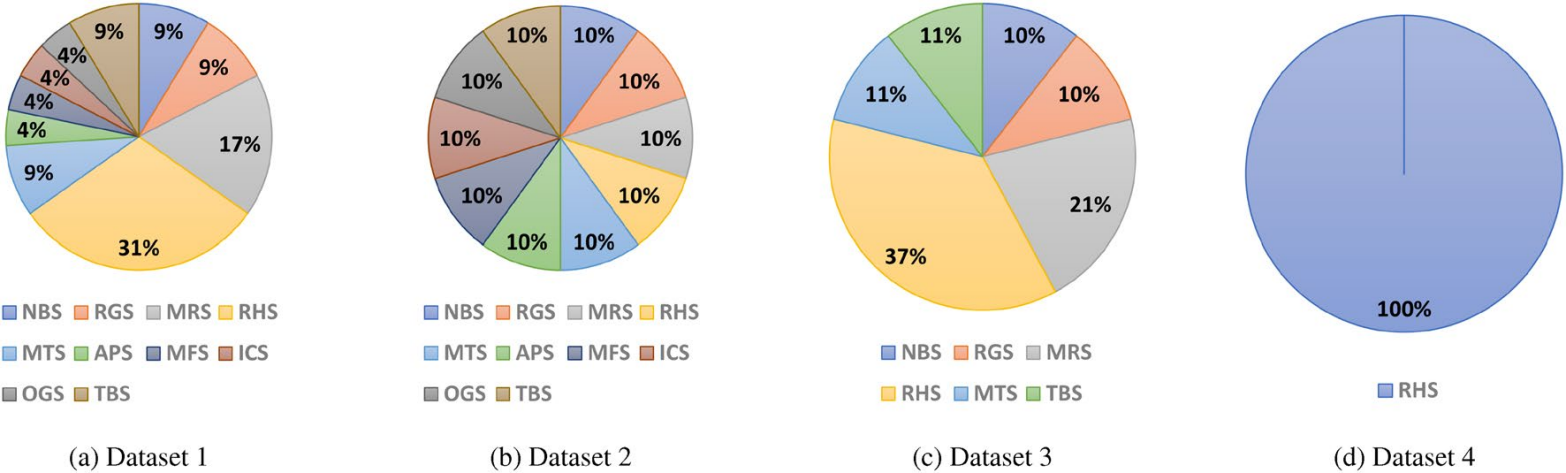
Distribution of superclass samples in different datasets.

Dataset 1 contains all the electrical data involved, and we conduct model training based on this dataset and use it as a benchmark. The function or type of each appliance in dataset 2 has certain differences. Furthermore, we increased the proportion of similar appliances in dataset 3. Finally, we set extreme conditions in dataset 4 with only resistive heating appliances. Figure 3 shows the ratio of the number of electrical samples in different superclasses.

The subgraphs (a)–(d) in the Fig. 3 represent the proportion of the total number of electrical samples of each superclass in the dataset 1-4. Subgraph (a) shows the overall distribution of the benchmark dataset. It can be seen from the subgraphs (b)-(d) that as the label of the dataset increases, the proportion of similar appliances gradually increases until only one superclass of appliances is included.

In this paper, experiments are conducted on both traditional VI trajectory mapping and structured VI trajectory mapping methods using AlexNet, lightweight AlexNet, and SVM. Since the distribution of sample sizes in different load categories is balanced, only accuracy and average macro-F1 indicators are compared. In addition, to compare the complexity of the methods, this paper also compares the training time per epoch (during which samples are transformed from raw data to corresponding feature maps in real-time), model parameter quantity, and computational complexity.

### Parameter settings

The structure of the neural network designed in this paper is shown in Table 2. The experiment is based on Pytorch, using softmax as the classifier and cross-entropy for loss calculation. The network model is optimized by the SGD optimizer, the momentum is set to 0.9, the L2 regularization parameter is set to 2e-4, the initial learning rate is set to 0.01, the learning rate decays to the original 0.1 every 30 rounds of training, and the batch size is set to 128, for a total of 120 rounds of model training.

### Experiments on dataset 1

First, this paper constructs dataset 1 based on the sample data of all 23 types of loads participating in the experiment. Since the distribution of sample data of each load is balanced, this paper only needs to compare the accuracy without additional F1 evaluation. In order to reflect the generalization performance of the method, the datasets used in this paper are evaluated by 5-fold cross-validation. We divide the dataset into five equal parts, four of which are used for model training, and one is used to verify and select the best model. After training, select the model with the best performance on the validation set, verify the test set and output the results. Finally, we averaged the five test results, and calculated the mean value of the identification accuracy of each class, the accuracy of the class with the worst identification effect, and the accuracy rate of the class with the best identification effect. The statistics are shown in Table 3.

**Figure 4:**
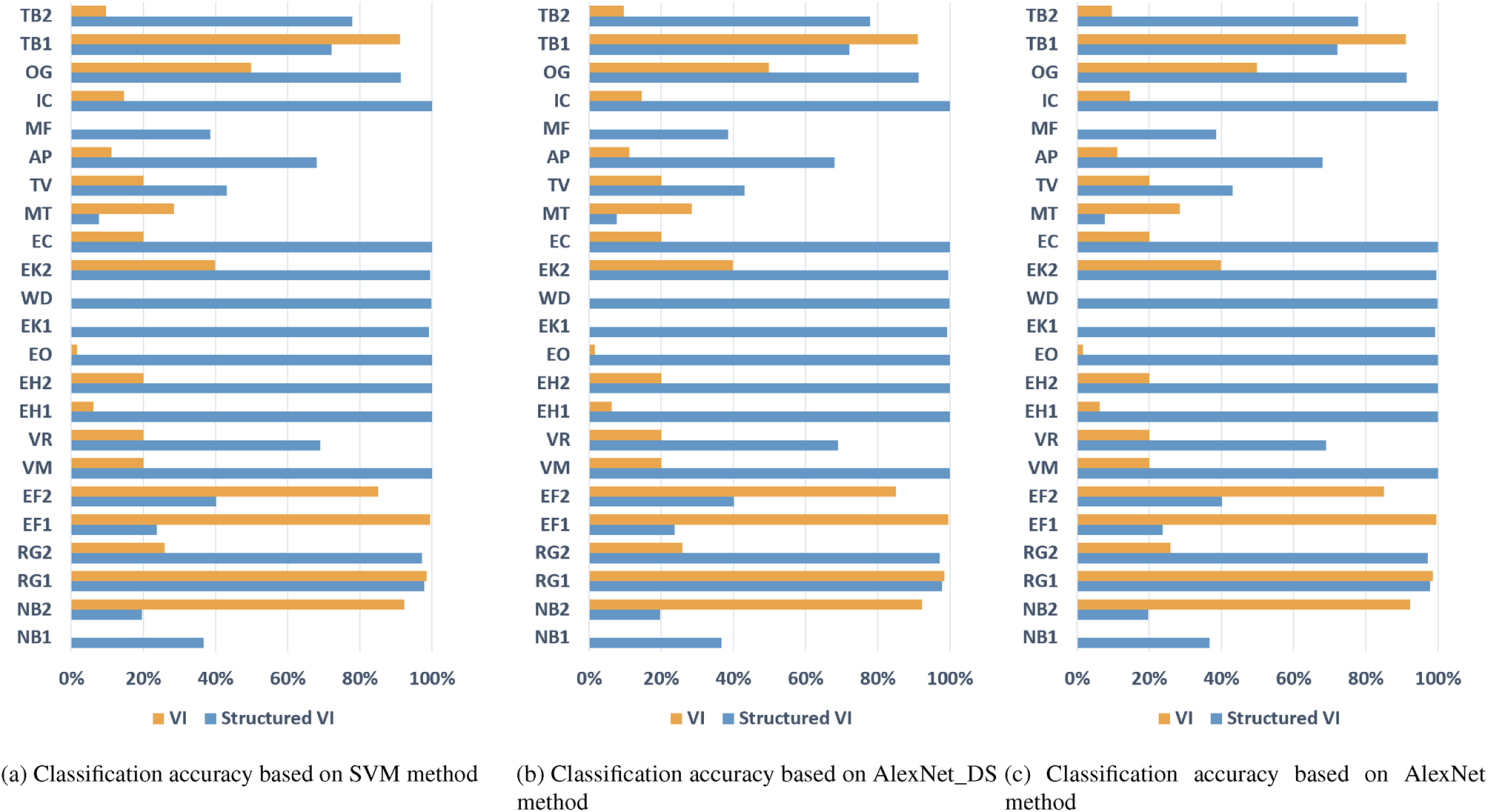
Experimental results on dataset 1.

In Table 3, accuracy represents the average recognition accuracy of each class, Macro-F1 represents the average of the Macro-F1 values of each class, and Time represents the average training time per epoch. From the experimental results in Table 3, the proposed structured V-I mapping method shows better performance and robustness compared to the VI trajectory mapping method in different models. Figures 4a–c show the recognition accuracy of each class under different model conditions. It can be seen that the recognition performance of the proposed method is better than the traditional V-I method in most classes and has relatively balanced performance.

**Table 3:** Accuracy comparison on the test set.

Model	Method	Accuracy(%)	Macro-F1(%)	Time(s)
SVM	V-I	32.75	28.51	35.94
	Ours	73.06	71.91	20.66
AlexNet-DS	V-I	94.32	94.11	41.68
	Ours	99.52	99.52	21.27
AlexNet	V-I	94.57	94.61	52.23
	Ours	99.7	99.7	30.29

### Experiments on dataset 2

**Figure 5:**
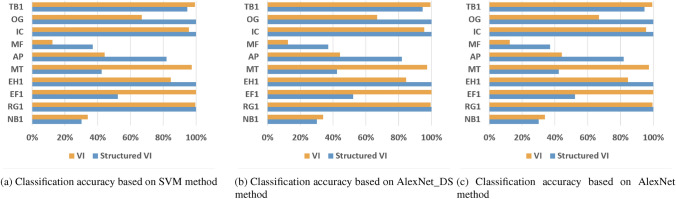
Experimental results on dataset 2.

In order to test the performance of the structured mapping method in a scene without similar appliances, we extract 10 different types of appliances for experiments. The experimental results are shown in Table 4. As shown in Figs. 5a–c, in terms of the recognition performance of each class, the structured V-I mapping method is generally better than the traditional V-I trajectory mapping method, except for some slight differences in individual classes in the SVM method.

**Table 4:** Accuracy comparison on the test set.

Model	Method	Accuracy(%)	Macro-F1(%)	Time(s)
SVM	V-I	73.34	70.01	16.28
	Ours	73.64	71.14	10.03
AlexNet-DS	V-I	99.73	99.73	19.33
	Ours	99.75	99.75	10.33
AlexNet	V-I	99.77	99.77	23.33
	Ours	99.79	99.79	14.31

### Experiments on dataset 3

**Figure 6:**
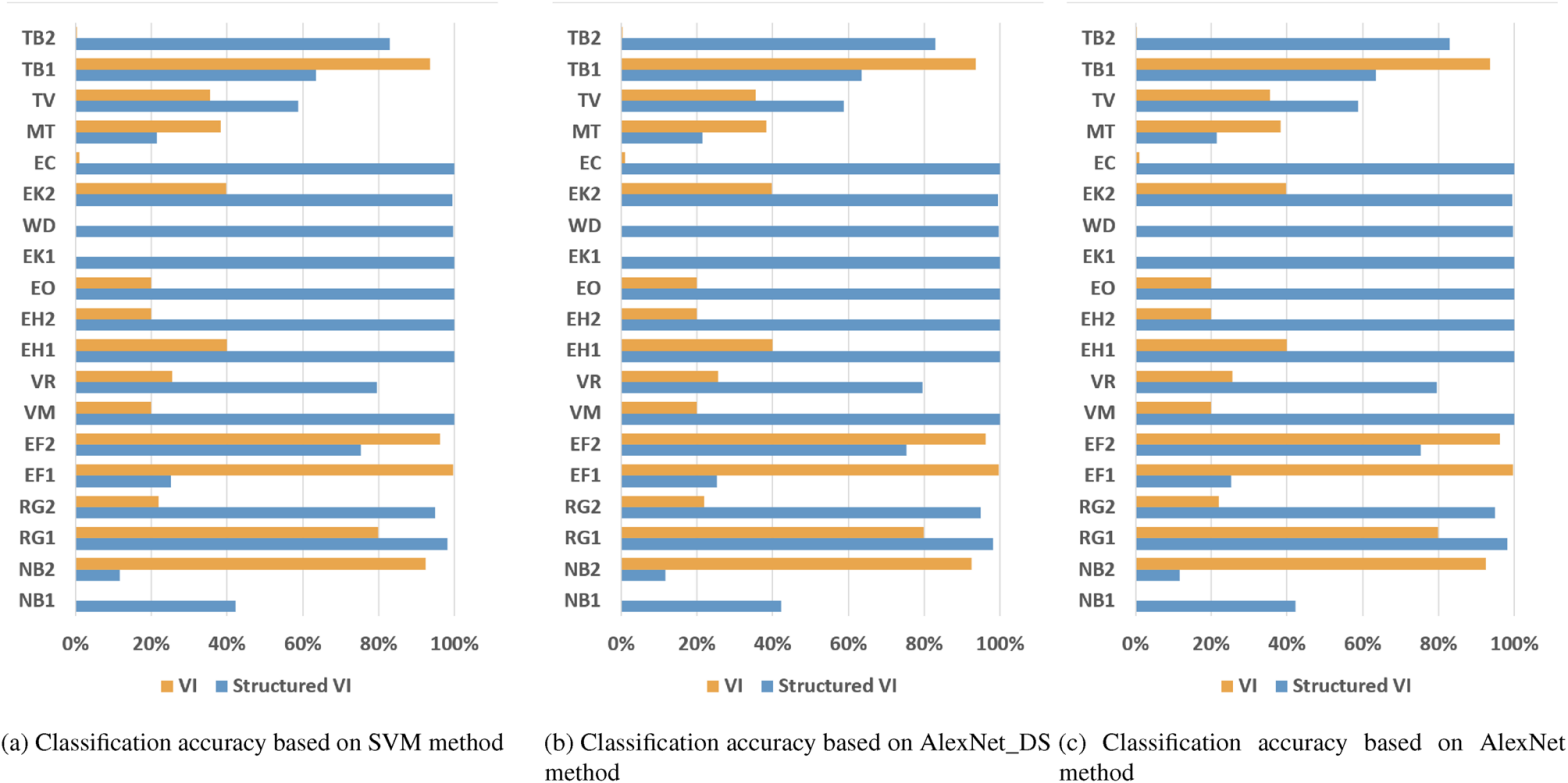
Experimental results on dataset 3.

Furthermore, this paper collects classes with similar electrical appliances into dataset 3. In dataset 3, each load can find at least one type of load with similar current. Table 5 shows that the overall performance of the traditional V-I trajectory mapping method is poor on this dataset. In addition, the results in Figs. 6a–c show that the VI trajectory-based method cannot effectively distinguish at least one class of two similar appliances. Therefore, it can be preliminarily inferred that with the increase of similar appliances in the training set, the structured V-I mapping method exhibits more stable performance.

**Table 5:** Accuracy comparison on the test set.

Model	Method	Accuracy(%)	Macro-F1(%)	Time(s)
SVM	V-I	38.1	34.44	29.71
	Ours	76.45	75.15	17.87
AlexNet-DS	V-I	92.28	92.4	36.29
	Ours	99.65	99.63	18.29
AlexNet	V-I	93.31	93.41	45.16
	Ours	99.67	99.67	25.78

### Experiments on dataset 4

**Figure 7:**
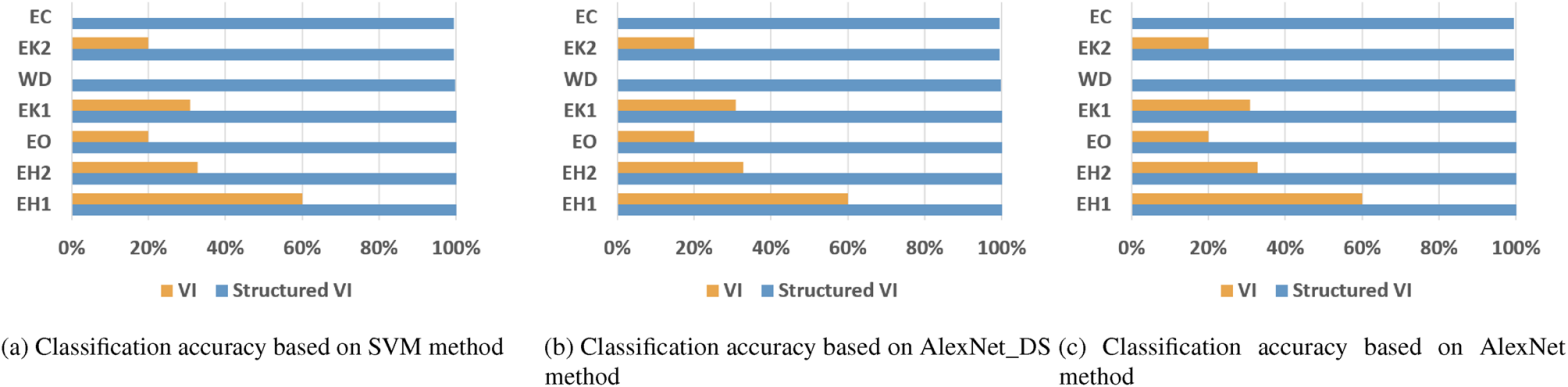
Experimental results on dataset 4.

Since there is no harmonic signal in resistive heating appliances, the current waveform between different appliances is highly consistent. In the last group of experiments in this paper, a dataset with resistive elements as the main components of the load is designed. Furthermore, as shown in Figs. 7a–c and Table 6, when there are fewer appliance types and more similar appliance types, the structured V-I mapping method can still exhibit stable performance and effectively model the data using simple classifiers, demonstrating the advantage of this method’s feature representation. In contrast, the V-I trajectory mapping method shows unstable performance.

**Table 6:** Accuracy comparison on the test set.

Method	Method	Accuracy(%)	Macro-F1(%)	Time(s)
SVM	V-I	23.37	21.95	11.15
	Ours	99.81	99.81	7.23
AlexNet-DS	V-I	81.98	81.98	13.69
	Ours	99.98	99.98	7.37
AlexNet	V-I	82	82.08	16.37
	Ours	99.98	99.98	10.28

In terms of the time complexity of model training, although the method in this paper requires more dimensional transformations than the traditional V-I trajectory mapping, the structured V-I mapping method directly maps the structural relationship of voltage and current to the two-dimensional array of the corresponding channel, so it will have higher conversion efficiency. However, the traditional V-I trajectory method needs to map the current and voltage into images, which will lead to more time loss in the conversion process. This can be easily seen from the average training time, where although the proposed method has higher dimensionality, it still has a longer average training time compared to the V-I trajectory mapping method, which requires converting images. In addition, this paper analyzed the parameter and computational costs of several different models based on different methods, as shown in the Table 7. It can be seen that although the structured trajectory mapping method has a small increase in parameter and computational costs compared to the V-I trajectory mapping method, this difference has almost negligible negative effects in neural networks. While the difference in SVM is relatively large, its parameter count mainly depends on the number of dimensions of the input features, and in most scenarios, the performance of SVM methods is not satisfactory. Therefore, the comparison of parameter and computational costs in SVM is less meaningful. In summary, compared to the traditional V-I trajectory mapping method, the actual computational complexity of the proposed structured V-I trajectory mapping method is almost negligible.

**Table 7:** Comparison between the model parameters and FLOPs.

Model	Method	Parameters(K/M)	FLOPs(K/M)
SVM	V-I	147.20K	147.22K
	Ours	441.60K	441.62K
AlexNet-DS	V-I	303.13M	45.35M
	Ours	304.97M	45.34M
AlexNet	V-I	32.43M	8.33M
	Ours	32.67M	8.33M

## Results and discussion

In order to realize the load identification technology of more refined classification, improve the identification efficiency and robustness of NILM. This paper proposes a load identification method based on structured V-I mapping, which is inspired by V-I trajectory mapping. At the same time, in order to solve the limitations of traditional V-I trajectory mapping methods in terms of conversion efficiency and identification of similar electrical appliances, we creatively map the point-to-point relationship characteristics of periodic voltage and current directly into the two-dimensional array of different channels, and design a neural network model to train the classification model. The experimental results show that the structural V-I mapping method combined with neural network training model designed in this paper outperforms the traditional V-I trajectory mapping method in various scenarios. In addition, compared with the existing V-I trajectory mapping methods, the proposed method in this paper has two novel viewpoints as follows:

1. In this paper, the point-to-point multiplication, addition, and subtraction of periodic voltage and periodic current are directly mapped to three-dimensional tensor, and then the model is trained in a manner similar to image classification. Applying this idea, more channels can be extended to store more load characteristics for the neural network to automatically extract effective features, such as weighted mapped currents to highlight harmonic characteristics, autocorrelation characteristics between currents, etc.

2. This paper focuses on the load identification method based on structured V-I mapping, but at the same time, the event detection method is also a crucial technology in NILM, and its weak target identification and identification under the influence of complex background noise are still facing challenges. The proposed method in this paper can map the voltage and current data of time series to multiple channels. It will also be an interesting direction to perform refined switching event detection combined with timing analysis.

This paper provides a novel input representation method in the NILM load identification method, and verifies the effectiveness of the proposed method through relevant experiments. In future research, we will further search for a better representation combination, optimize the network model at the same time, reduce parameters and improve computing efficiency. In this way, the neural network model with high resource and high computing requirements can be deployed to the embedded terminal with limited performance to achieve real-time monitoring and improve the engineering practical performance of NILM.

## Data Availability

The datasets generated and/or analyzed during the current study are available in the DataSet_SEIN_1 repository, https://github.com/duzehua/DataSet_SEIN_1.
